# Breaking the Cross-Sensitivity Degeneracy in FBG Sensors: A Physics-Informed Co-Design Framework for Robust Discrimination

**DOI:** 10.3390/s26020459

**Published:** 2026-01-09

**Authors:** Fatih Yalınbaş, Güneş Yılmaz

**Affiliations:** Faculty of Engineering, Department of Electrical and Electronics Engineering, Bursa Uludağ University, 16059 Bursa, Türkiye; gunesy@uludag.edu.tr

**Keywords:** fiber bragg grating, cross-sensitivity, physics-informed neural networks, transfer matrix method, sensor fusion

## Abstract

The simultaneous measurement of strain and temperature using Fiber Bragg Grating (FBG) sensors presents a significant challenge due to the intrinsic cross-sensitivity of the Bragg wavelength. While recent studies have increasingly employed “black-box” machine learning algorithms to address this ambiguity, such approaches often overlook the physical limitations of the sensor’s spectral response. This paper challenges the assumption that advanced algorithms alone can compensate for data that is physically ambiguous. We propose a “Sensor-Algorithm Co-Design” methodology, demonstrating that robust discrimination is achievable only when the sensor architecture exhibits a unique, orthogonal physical signature. Using a rigorous Transfer Matrix Method (TMM) and 4 × 4 polarization analysis, we evaluate three distinct architectures. Quantitative analysis reveals that a standard Quadratically Chirped FBG (QC-FBG) functions as an “ill-conditioned baseline” failing to distinguish measurands due to feature space collapse ($K_{cond}>4600$). Conversely, we validate two robust co-designs: (1) An Amplitude-Modulated Superstructure FBG (S-FBG) paired with an Artificial Neural Network (ANN), utilizing thermally induced duty-cycle variations to achieve high accuracy (~3.4 °C error) under noise; and (2) A Polarization-Diverse Inverse-Gaussian FBG (IG-FBG) paired with a 4 × 4 K-matrix, exploiting strain-induced birefringence ($K_{cond}\approx 64$). Furthermore, we address the data scarcity issue in AI-driven sensing by introducing a Physics-Informed Neural Network (PINN) strategy. By embedding TMM physics directly into the loss function, the PINN improves data efficiency by 2.2× compared to standard models, effectively bridging the gap between physical modeling and data-driven inference, addressing the critical data scarcity bottleneck identified in recent optical sensing roadmaps.

## 1. Introduction

The trajectory of modern engineering, encompassing critical sectors such as aerospace, civil infrastructure, energy systems, and biomedical devices, is increasingly defined by the integration of “smart” capabilities and the transition towards autonomous operation. At the center of this paradigm shift lies the urgent need for real-time, distributed sensing networks capable of monitoring the structural integrity and operational parameters of complex systems with high fidelity. This evolution from passive design to active, Condition-Based Maintenance (CBM) requires a sensory nervous system that conventional technologies struggle to provide. In this context, Fiber Bragg Grating (FBG) sensors have emerged not merely as an alternative, but as the primary candidate for next-generation sensing, progressively replacing traditional electromechanical measuring devices (such as resistive strain gauges and thermocouples) due to their intrinsic advantages in multiplexing, durability, and signal integrity in harsh environments.

As extensively detailed in the seminal review by Kersey et al. [1], FBG sensors offer a unique combination of features: they are passive, electrically non-conductive, immune to electromagnetic interference (EMI), and chemically inert. These characteristics make their deployment indispensable in harsh conditions where traditional sensors would fail, such as the high-voltage windings of transformers, fuel tanks of aircraft, or corrosive environments of petrochemical pipelines. Furthermore, the multiplexing capability of FBGs, which allows dozens of sensors to be inscribed onto a single optical fiber, enables the creation of dense, quasi-distributed sensor networks capable of mapping strain and temperature fields with high spatial resolution.

Recent developments have further expanded the application spectrum of FBGs. For instance, Risi et al. [2] highlighted the transformative potential of fiber optic sensors in biomedical applications, ranging from smart catheters to wearable health monitors, where the miniature form factor and biocompatibility of silica glass are vital. Similarly, Majumder et al. [3] drew attention to the critical role of FBGs in the field of Structural Health Monitoring (SHM), particularly for the early detection of fatigue cracks and load distributions in aging civil infrastructure.

Despite these significant advantages, the practical effectiveness of FBG sensors is fundamentally constrained by a critical physical limitation known as “cross-sensitivity” [4]. The operating principle of a standard FBG is based on the reflection of a specific wavelength of light ($\lambda_{B}$) that satisfies the Bragg condition:

(1) $$\lambda_{B}={2n}_{eff}\Lambda$$

Here, $n_{eff}$ represents the effective refractive index of the fiber core, and Λ represents the period of the grating modulation. The sensor acts as a spectral filter, reflecting a narrow bandwidth centered at $\lambda_{B}$ while transmitting all other wavelengths.

The problem arises from the fact that these two governing parameters, $n_{eff}$ and Λ, are simultaneously affected by both mechanical strain (ϵ) and temperature (T). As explained by Jones 4, a shift in the reflected wavelength (${\Delta \lambda }_{B}$) is a superposition of the thermo-optic effect (change in refractive index with temperature), thermal expansion (change in grating period with temperature), and the elasto-optic effect (change in index and period with strain). Mathematically, this relationship is expressed as:

(2) $${∆\lambda }_{B}=\lambda_{B}\left(1-p_{e}\right)\epsilon +\lambda_{B}\left(\alpha_{\Lambda }+\xi \right)∆T$$

where $p_{e}$ denotes the effective photo-elastic coefficient, $\alpha_{\Lambda }$ the thermal expansion coefficient of the fiber, and ξ the thermo-optic coefficient.

In a dynamic operational environment where both temperature and strain fluctuate simultaneously—for example, an aircraft wing exposed to aerodynamic loading while experiencing rapid altitude-induced temperature changes—a single ${∆\lambda }_{B}$ measurement is mathematically insufficient to determine the two unknowns (T and ϵ). This ambiguity leads to an ill-posed inverse problem. Without a secondary source of information, it is impossible to distinguish whether a 1 nm wavelength shift is caused by a significant mechanical load or a moderate temperature increase. This “blind spot” in sensing physics compromises the reliability of FBG data and has historically necessitated the use of cumbersome compensation techniques.

The engineering community has acknowledged the cross-sensitivity problem for decades, leading to a proliferation of proposed solutions. These methodologies can generally be categorized as hardware-focused approaches aimed at physically separating the measurands, and software-focused approaches attempting to separate them post-measurement using algorithms. However, a critical review of the literature reveals that existing solutions often trade one set of limitations for another, failing to provide a robust, scalable, and cost-effective answer.

The earliest and most intuitive attempts to resolve cross-sensitivity relied on hardware redundancy. The basic rationale of these approaches is to generate a system of two equations for two unknowns (T and ϵ) by adding a second sensor element with a different response coefficient.

Xu et al. [5] pioneered the dual-wavelength strategy by superimposing two FBGs with widely separated Bragg wavelengths in the same optical fiber, demonstrating that a theoretically solvable matrix equation could be obtained. The mismatch in dispersion characteristics at different wavelengths results in slightly different sensitivity coefficients for strain and temperature. However, as subsequent analyses have shown, the difference in coefficients between the two silica-based gratings is often very small. This leads to a sensing matrix with a very high condition number, meaning that even small amounts of measurement noise from the optical interrogator translate into massive errors in the final calculation.

To improve the conditioning of the sensing matrix, researchers have investigated hybrid sensor configurations. Patrick et al. [6] proposed a hybrid Fiber Bragg Grating/Long Period Grating (LPG) sensor. LPGs exhibit significantly higher sensitivity to temperature and refractive index changes compared to standard FBGs, thus providing the necessary orthogonality in the sensitivity matrix. While scientifically sound, this approach introduces serious practical disadvantages. LPGs are significantly longer than FBGs (typically several centimeters vs. millimeters), are highly sensitive to bending and transverse loads, and possess broader spectral resonances that are harder to monitor with high precision. These factors complicate sensor packaging and reduce the spatial resolution of the system.

The pursuit of orthogonality has driven the development of sophisticated hardware configurations. Beyond the extrinsic mechanical packaging solutions like the cantilever beams proposed by Li et al. [7] or the vibration-isolating ‘piston’ structures developed by Lu et al. [8], researchers have explored intrinsic optical mechanisms. Ding et al. [9] demonstrated that inserting high-birefringence fibers into a Sagnac interferometer loop could generate a differential phase response, effectively decoupling strain and temperature with high precision. Similarly, Zou et al. [10] exploited the unique spectral characteristics of Brillouin dynamic gratings, which create independent frequency shifts for different measurands. While these interferometric and scattering-based methods achieve impressive discrimination, they invariably increase system complexity, require bulky interrogators, and compromise the multiplexing scalability that defines the FBG advantage. This necessitates a solution that retains the simplicity of a standard FBG while introducing the necessary physical dimensionality.

In response to the physical limitations of hardware redundancy, the research focus has shifted dramatically in recent years toward software-driven solutions, specifically the use of Machine Learning (ML) and Artificial Intelligence (AI). The premise is compelling: instead of modifying the sensor, Deep Learning (DL) can be used to extract subtle, non-linear correlations from the sensor’s spectrum that traditional linear algebra cannot capture.

Sarkar et al. [11] were among the first researchers to rigorously apply this concept to single FBG discrimination. In their landmark work, Sarkar et al. [11] demonstrated that the reflectivity of side lobes in a standard FBG spectrum is not perfectly symmetric, and this asymmetry evolves differently under strain and temperature fields. By training a machine learning model on the power of these side lobes, Sarkar et al. [11] achieved a discrimination accuracy of approximately 90%. This work was pivotal in shifting the community’s attention from mere peak tracking ($\lambda_{B}$ measurement) to full-spectrum analysis.

Following this trajectory, Li et al. [12] and Hameed et al. [13] proposed increasingly complex Deep Learning architectures, including Convolutional Neural Networks (CNN) and Long Short-Term Memory (LSTM) networks, to process raw spectral data of FBGs. Li et al. [12] argued that deep networks could automatically learn features invisible to the human eye, potentially providing a “universal” solution to cross-sensitivity without the need for special grating structures.

A significant recent contribution to this field was provided by Choi et al. [14], who combined high-speed Wavelength Swept Lasers (WSL) with Machine Learning. Choi et al. [14] addressed the data acquisition bottleneck by enabling the collection of massive datasets (over 46,000 samples) in the temporal domain using a Fourier Domain Mode Locked (FDML) laser and a Polygon-Scanner Filter. Feeding six specific variables—peak voltages and time positions of the main peak and side lobes—into a Scikit-learn-based regressor, Choi et al. [14] reported a simultaneous measurement accuracy of 95%. Their work highlighted the potential of high-speed interrogation in capturing dynamic variations that static Optical Spectrum Analyzers (OSA) might miss.

While data-driven models proposed by Li et al. [12] and Choi et al. [14] represent a paradigm shift, they suffer from a fundamental ‘physics-blindness’. These models treat optical spectra as arbitrary statistical distributions, ignoring the governing Maxwell’s equations. To bridge this gap, the domain of Scientific Machine Learning (SciML) has introduced Physics-Informed Neural Networks (PINNs). As reviewed by Wang et al. [15], PINNs have recently been adopted in fiber optics to solve inverse problems where data is scarce. Notable applications include the prediction of ultrafast nonlinear dynamics [16] and mode characterization in quantum communication networks [17]. Most relevant to this work, Li et al. [18] recently proposed a PINN framework for dual-parameter decoupling using dynamic weight assignment to suppress crosstalk. However, existing approaches largely focus on optimizing the algorithm to solve a fixed sensor problem. Our work diverges by proposing a ‘Co-Design’ strategy: we optimize the sensor physics to create a distinct signature (Co-Design I & II) that is inherently tailored for efficient decoding by a physics-constrained algorithm.

However, a critical theoretical examination reveals a fundamental flaw in this “black box” trend. Although Choi et al. [14] and Sarkar et al. [11] achieved admirable empirical results, their success largely relies on the presence of secondary spectral features (like side lobes) that are often uncontrolled artifacts of the manufacturing process rather than designed features. A standard, non-apodized FBG exhibits side lobes due to the abrupt termination of index modulation at the fiber ends. As noted by Erdogan [19], the amplitude and position of these lobes are highly sensitive to minute variations in production.

Consequently, an ML model trained on a specific FBG, as done by Choi et al. [14] and Sarkar et al. [11], would likely fail when applied to a second FBG, even if nominally identical. The “features” learned by the AI are not fundamental physical responses to Temperature and Strain, but rather the unique “fingerprint” of that specific grating’s imperfections. This lack of generalizability is a fatal bottleneck for mass production. It is not feasible to train a separate neural network for every single sensor in a network of thousands.

Moreover, relying on standard FBGs assumes that the spectrum contains the necessary information. While standard Deep Learning has shown promise, it fundamentally ignores the governing physical laws of light propagation, treating the sensor merely as a statistical generator. To address this limitation, the field has recently seen the emergence of Physics-Informed Neural Networks (PINNs). Wang et al. [15] provided a comprehensive review of PINN applications in optical fibers, highlighting their potential to solve inverse problems with limited data. Recently, Li et al. [18] successfully applied a PINN framework for dual-parameter decoupling by assigning dynamic physical weights, demonstrating superior crosstalk suppression. Similarly, Zhang et al. [17] extended PINN architectures to multimode fibers in quantum communication contexts. However, these studies primarily focus on algorithmic optimization. In contrast, our work proposes a holistic ‘Co-Design’ strategy where the sensor architecture itself is optimized to aid the PINN, rather than relying on the network to compensate for ill-conditioned physical designs. Our analysis challenges this assumption. As highlighted in recent reviews by Avellar et al. [20] and Zhang et al. [21], if the sensor physics dictates that Strain and Temperature cause linearly dependent spectral shifts, no amount of algorithmic complexity can separate them without overfitting to noise. As we will show later in this report, a standard Quadratically Chirped FBG (QC-FBG) presents a feature space that collapses onto a 1D manifold. In such a scenario, the “intelligence” of the algorithm is irrelevant; the information is simply not there. Chethana, et al. [22] and Uduagbomen et al. [23] further emphasize that standard deep learning models often suffer from overfitting when applied to such noisy, high-dimensional spectral data typical of optical interrogators.

**Figure 1:**
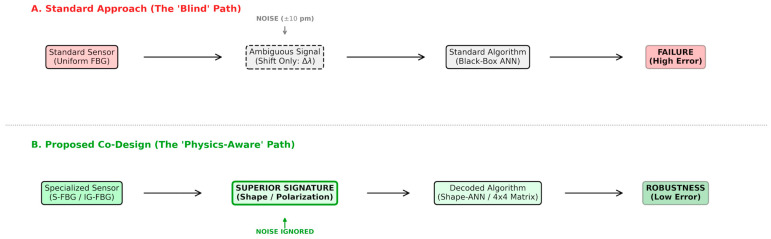
The conceptual framework of Sensor-Algorithm Co-Design. While the standard approach (**A**) suffers from signal ambiguity, the proposed co-design (**B**) engineers the sensor physics to provide orthogonal signatures decodable by specialized algorithms.

Therefore, the limitations of existing solutions can be summarized as a dilemma: Hardware solutions offer physical robustness but lack scalability and compactness; existing Software/ML solutions offer scalability but lack physical robustness and generalizability. We argue that the way forward lies in the convergence of these two domains: Sensor-Algorithm Co-Design. As illustrated in Figure 1, the standard approach often leads to failure when the physical signal is ambiguous, whereas our proposed framework engineers the signal to be inherently decodable.

In this paper, we propose a “Sensor-Algorithm Co-Design” framework to address these challenges. We argue that robust sensing cannot be achieved by algorithmic complexity alone; it requires designing the sensor’s physical signature (hardware) in tandem with the decoding algorithm (software). We support this thesis through a comprehensive in silico investigation that contributes to the field in three specific ways:

- Quantitative Failure Analysis of Baseline Sensors: We provide a rigorous failure analysis of standard broad-spectrum sensors (QC-FBG). While recent studies such as those by Li et al. [12] and Hameed et al. [13] suggest that deep learning can extract parameters from complex spectra, our results demonstrate that QC-FBGs represent an “ill-conditioned” system where temperature and strain responses remain linearly dependent ($K_{cond}>4600$). This proves that without physical orthogonality, advanced ANNs fail to converge, confirming the limitations of purely data-driven approaches in ambiguous physical domains.
- Validation of Orthogonal Architectures: We validate two novel robust architectures—the Amplitude-Modulated S-FBG and the Polarization-Diverse IG-FBG—that introduce specific physical mechanisms to break cross-sensitivity degeneracy. Unlike the approach of Choi et al. [14], which relies on manufacturing artifacts (side-lobes) and requires massive datasets (46,000 samples), our designs leverage deterministic superstructure concepts [24] and inverse-Gaussian apodization profiles [25] to create distinct, physically orthogonal feature spaces decodable with high accuracy (~3.4 °C error).
- Data Efficiency via Physics-Informed Learning: We introduce a Physics-Informed Neural Network (PINN) strategy to overcome the “data scarcity” bottleneck identified in recent reviews by Avellar et al. [20] and Zhang et al. [21]. By embedding the Transfer Matrix Method (TMM) physics [19,26] directly into the network’s loss function, we demonstrate that the model can be trained effectively using primarily unlabeled data. This approach improves data efficiency by 2×2 compared to standard models, bridging the gap between physical modeling and data-driven inference as pioneered by Raissi et al. [27] and recently adapted for optical systems [28].

## 2. Materials and Methods

To overcome the limitations described above, we propose a holistic design philosophy where the physical architecture of the sensor is specifically engineered to generate orthogonal features optimized for solving by a corresponding algorithm. To validate this approach, we employ a rigorous “in silico” (computer simulation) validation framework combining forward physical modeling with advanced inverse solvers. The numerical results and performance metrics reported in this study were generated using Python 3.10 environment, utilizing the PyTorch version 2.3.1 library for deep learning implementations and NumPy version 1.26.4 for matrix operations.

### 2.1. Forward Modeling: Transfer Matrix Method (TMM)

The foundation of our analysis is the accurate simulation of light-matter interaction within the optical fiber. We utilize the Transfer Matrix Method (TMM) based on Coupled Mode Theory, as formulated by Erdogan [19]. This method allows for the precise calculation of the spectral response of complex grating structures with arbitrary refractive index profiles.

The grating is conceptualized as a sequence of N uniform segments. For each segment i, the relationship between the forward-propagating field ($E_{f}$) and the backward-propagating field ($E_{b}$) is governed by a 2×2 transfer matrix $F_{i}$:

(3) $$\left[\begin{array}{c} E_{f}\left(z_{i}\right)\\ E_{b}\left(z_{i}\right) \end{array}\right]\;=\;F_{i}\left[\begin{array}{c} E_{f}\left(z_{i-1}\right)\\ E_{b}\left(z_{i-1}\right) \end{array}\right]$$

where the matrix $F_{i}$ is defined as:

(4) $$F_{i}\;=\;\left[\begin{array}{cc} \cos h\left(\gamma dz\right)-i\hat{\sigma }\frac{\sin h\left(\gamma dz\right)}{\gamma } & -i\kappa \frac{\sin h\left(\gamma dz\right)}{\gamma }\\ i\kappa \frac{\sin h\left(\gamma dz\right)}{\gamma } & \cosh\left(\gamma dz\right)+\hat{i\sigma }\frac{\sin h\left(\gamma dz\right)}{\gamma } \end{array}\right]$$

Here, dz is the segment length, κ is the AC coupling coefficient ($\kappa \;=\;\pi \Delta n\left(z\right)/\lambda$) determined by the modulation depth, and $\hat{\sigma }$ is the DC self-coupling coefficient. The parameter γ represents the coupling strength, defined as $\gamma \;=\;\sqrt{\kappa^{2}-{\hat{\sigma }}^{2}}$. Environmental perturbations modify these parameters via the thermo-optic (ξ) and photo-elastic ($p_{e}$) coefficients:

(5) $$n_{eff}\left(T\right)\;=\;n_{0}\;+\;\xi \Delta T$$

(6) $$\Lambda \left(T,\epsilon \right)=\Lambda_{0}\left(1+\alpha \Delta T+\epsilon \right)$$

(7) $$n_{eff}\left(\epsilon \right)=n_{eff}\left(T\right)\;\cdot \;\left(1-p_{e}\epsilon \right)$$

For the IG-FBG, we extended this to a 4 × 4 Vectorial TMM [14] to model strain-induced birefringence ($B\;=\;n_{y}\;-{\;n}_{x}$).

To simulate the sensor’s response to environmental perturbations, the TMM parameters are updated dynamically. The effective refractive index $n_{eff}$ and grating period Λ are modified according to the thermo-optic and thermal expansion coefficients for temperature (T), and according to the photo-elastic and mechanical elongation effects for strain (ϵ).

For the analysis of polarization effects in the IG-FBG architecture, we extend the standard scalar TMM to a 2×2 Vectorial TMM, as derived by Erdogan and Mizrahi [14]. This extension allows us to independently model the evolution of two orthogonal polarization modes (x and y) and the coupling between them induced by birefringence caused by transverse or axial loads.

The Transfer Matrix Method (TMM) provides the necessary numerical flexibility for this study. While analytic solutions based on coupled-mode theory, such as those derived by Liau et al. [29] using eigenvalue and eigenvector techniques, offer computational efficiency for uniform gratings, they cannot easily accommodate the complex, spatially varying refractive index profiles (e.g., Inverse-Gaussian apodization) or the non-uniform strain fields analyzed here. Furthermore, to capture the strain-induced birefringence effects in the IG-FBG architecture, we employ a 4 × 4 Vectorial TMM formulation extended from the principles outlined by Liu et al. [30] for twisted and anisotropic fibers. This allows for the rigorous calculation of the cross-coupling between $HE_{11}^{x}$ and $HE_{11}^{y}$ modes under axial perturbation.

### 2.2. Mathematical Definition of Robustness

To rigorously quantify the stability of the sensor response mentioned in the introduction, we utilize the Condition Number ($K_{cond}$) of the sensitivity matrix K. For a linearized system y=Kx, the condition number is defined via Singular Value Decomposition (SVD) as:

(8) $$K_{cond}\;=\;\frac{\sigma_{max}}{\sigma_{min}}$$

where $\sigma_{max}$ and $\sigma_{min}$ are the largest and smallest singular values of K. A sensor is defined as “Ill-Conditioned” if $K_{cond}$ is sufficiently large such that the error propagation creates a “Catastrophic Failure,” defined by the inequality:

(9) $$\frac{\left\|\delta x\right\|}{\left\|x\right\|}\leq K_{cond}\frac{\left\|\delta y\right\|}{\left\|y\right\|}$$

where $\frac{\left\|\delta x\right\|}{\left\|x\right\|}$ is the relative error in the output (calculated value) and $\frac{\left\|\delta y\right\|}{\left\|y\right\|}$ is the relative error in the input (measured value).

For our baseline QC-FBG, our simulations (see Section 3.1) reveal a $K_{cond}\approx 1.5\;\times \;10^{11}$, implying that the inverse problem is mathematically singular regardless of the algorithm used.

In this framework, a ‘Superior Physical Signature’ is defined as a hardware design that engineers the sensor’s spectral response to maintain a low $K_{cond}$ through feature space expansion, thereby preventing the ‘Catastrophic Failure’ of decoding algorithms when exposed to environmental noise.

### 2.3. Sensor Architectures Evaluated

**Figure 2:**
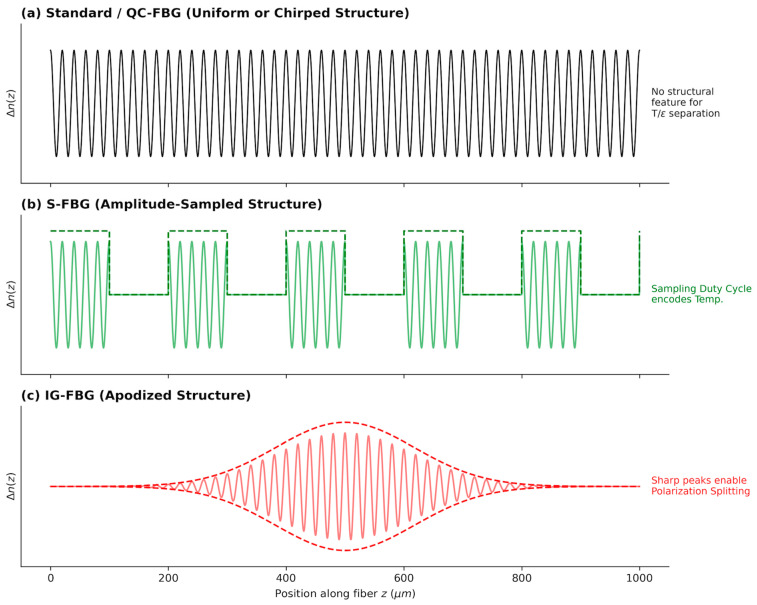
Visual representation of the refractive index modulation profiles along the fiber core for (**a**) the baseline QC-FBG, (**b**) the proposed S-FBG, and (**c**) the proposed IG-FBG.

To systematically evaluate the impact of physical design on algorithmic robustness, we modeled three distinct refractive index modulation profiles. The physical characteristics of these designs are visually compared in Figure 2, while their core mathematical parameters and physical signatures are summarized in Table 1. These architectures were selected to represent a progression from a standard broad-spectrum sensor to specialized designs engineered for feature orthogonality. These architectures represent an evolution from “ill-conditioned” (QC-FBG) to “ideal orthogonal” (IG-FBG). We evaluated three refractive index modulation profiles (Table 1).

#### 2.3.1. Baseline Reference: Quadratically Chirped FBG (QC-FBG)

The QC-FBG serves as our baseline, representing a common approach to increasing spectral information content. In this design, the grating period $\Lambda \left(z\right)$ varies non-linearly along the fiber axis z:

(10) $$\Lambda \left(z\right)\;=\;\Lambda_{0}\;+\;C{\;\cdot \;z}^{2}$$

where C is the chirp coefficient. This quadratic variation broadens the reflection bandwidth significantly compared to a uniform FBG. The prevailing assumption in the literature is that this “richer” broad spectrum contains more latent features for ML algorithms to utilize. However, physically, both temperature and strain act as global perturbations that shift the entire spectral envelope. We hypothesize that despite the increased bandwidth, the lack of localized, independent feature variations renders this architecture ill-conditioned for simultaneous sensing.

#### 2.3.2. Co-Design I: Amplitude-Modulated S-FBG

The first robust architecture proposed is the Superstructure FBG (S-FBG). Here, we introduce an intentional periodic sampling function $S\left(z\right)$ to the refractive index profile:

(11) $$∆n\left(z\right)\;=\;∆n_{std}\;\cdot \;S\left(z;D\right)$$

The parameter D represents the duty cycle of the sampling and $S\left(z\right)$ is a binary rectangular function with period P and duty cycle D.

**Figure 3:**
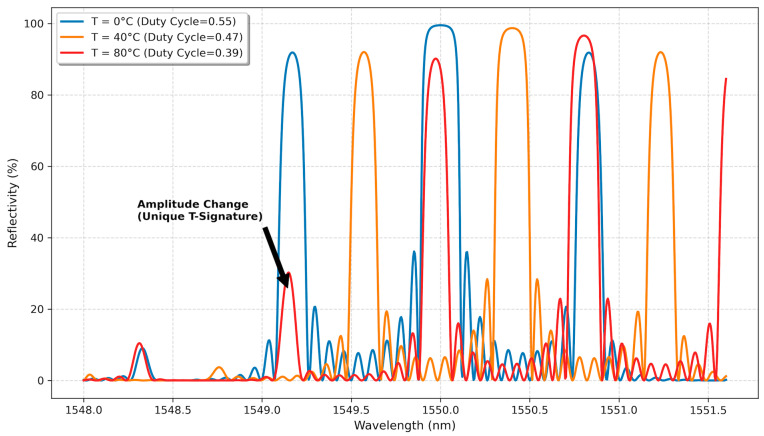
The “Superior Physical Signature” of the S-FBG. Simulations show that as temperature increases, the thermally induced change in the sampling duty cycle modulates the amplitude of the side-lobes, encoding temperature information orthogonally to the wavelength shift.

The “Co-Design” element involves the material engineering of the sensor as the introduction of a thermally responsive duty cycle. We model the FBG as being coated with or inscribed into a polymer with a high Thermal Expansion Coefficient (TEC), such as acrylate ($\alpha_{poly}>50\;\mu \epsilon /{}^{\circ}C$), significantly larger than that of the silica fiber ($\alpha_{SiO2}\approx 0.55\;\mu \epsilon /{}^{\circ}C$), as investigated by Guan et al. [24]. The thermal expansion mismatch between the polymer coating and the silica fiber induces a thermally dependent stress field that modulates the effective duty cycle D of the grating structure as temperature changes. As the temperature rises, the differential expansion of the polymer mask effectively modulates the duty cycle ($D\left(T\right)\;=\;D_{0}\;+\;\eta ∆T$). This physical mechanism encodes temperature information directly into the amplitude of the spectral side-lobes (as visualized in Figure 3), creating a secondary measurement channel orthogonal to the Bragg wavelength shift.

Orthogonality Mechanism:

- Strain primarily affects the Bragg wavelength $\lambda_{B}$ via the elasto-optic effect (Line Shift).
- Temperature affects $\lambda_{B}$ via the thermo-optic effect, but also alters the Duty Cycle D via polymer expansion.
- Critically, changes in Duty Cycle D alter the ratio of peak amplitudes between the main Bragg resonance and the sampling sidebands.

This creates a “Superior Physical Signature.” Temperature information is now encoded not just in the wavelength shift, but also in the amplitude modulation of the spectrum. As shown in our simulation results, this expands the data manifold from a 1D line to a separable 2D plane. The Neural Network can easily learn to read the “Amplitude” axis as a proxy for Temperature and the “Shift” axis as a proxy for Strain + Temperature, allowing for robust decoupling.

It should be noted that while this study models the polymer coating as a linear thermo-elastic material to establish the baseline efficacy of the co-design, real-world high-TEC polymers (such as acrylate or polyimide) exhibit viscoelastic properties. Future experimental iterations of this co-design will need to account for creep and relaxation effects, potentially by incorporating a time-dependent term into the PINN physics loss function.

#### 2.3.3. Co-Design II: Inverse-Gaussian Apodized FBG (IG-FBG)

The second proposed architecture, the IG-FBG, leverages polarization diversity. Standard FBGs often exhibit birefringence (refractive index difference between x and y axes), but the spectral peaks for the two polarizations usually overlap, blurring the signal.

We propose using an Inverse-Gaussian apodization profile characterized by $\Delta n\left(z\right)\;\propto exp\left(-\frac{{\left(z-L/2\right)}^{2}}{{2\omega }^{2}}\right)$. As analyzed by Lin et al. [25], this specific profile suppresses side lobes and, more importantly, produces ultra-narrow, sharp reflection peaks. Unlike standard apodization, which smooths the edges, the Inverse-Gaussian profile concentrates the index modulation index in the center of the grating while rapidly decaying towards the edges:

(12) $$\Delta n\left(z\right)\;=\;\Delta n_{max}\;\cdot \;exp\left(-\frac{{\left(z-L/2\right)}^{2}}{{2\omega }^{2}}\right)$$

The sharpness of these peaks is crucial for detecting strain-induced birefringence. Under axial strain, the cylindrical symmetry of the fiber core is broken, creating a refractive index difference between the x- and y-polarization axes ($n_{x}/n_{y}$). In a standard broad-peak FBG, this splitting is masked. In the IG-FBG, the sharp peaks allow the x- and y-modes to be spectrally resolved, providing a differential signal ($\Delta \lambda_{split}$) that is strictly proportional to strain and independent of temperature.

An Inverse-Gaussian apodized grating ($\Delta n\left(z\right)\propto e^{{-z}^{2}}$). The sharp spectral peaks allow for the detection of polarization splitting under strain. This profile can be achieved using standard phase-mask scanning techniques [25].

From a fabrication perspective, the IG-FBG does not require exotic materials or complex lithography. The inverse-Gaussian apodization profile can be achieved using standard phase-mask scanning techniques by spatially modulating the UV laser intensity or dithering the phase mask during the writing process. This compatibility with existing manufacturing infrastructure ensures that the proposed architecture is not merely a theoretical construct but a practical candidate for mass production.

Orthogonality Mechanism:

- Under Axial Strain, the cylindrical symmetry of the fiber is broken, inducing linear birefringence ($n_{x}\neq n_{y}$).
- The sharpness of the IG-FBG peaks allows this minute birefringence to be resolved as a spectral splitting of the Bragg peak into two distinct sub-peaks (x and y).
- Temperature shifts both peaks equally but does not cause significant splitting (in isotropic silica).

Thus, the “Splitting Distance” ($∆\lambda_{split}\;=\;\lambda_{y}\;-\;\lambda_{x}$) becomes a metric strictly specific to strain and independent of temperature. This transforms the discrimination problem from a complex non-linear regression into a simple linear system solvable by a 4×4 K-Matrix. The condition number for this architecture drops to 64.1, representing an improvement in robustness of nearly two orders of magnitude compared to the QC-FBG.

### 2.4. The Physics-Informed Neural Network (PINN)

**Figure 4:**
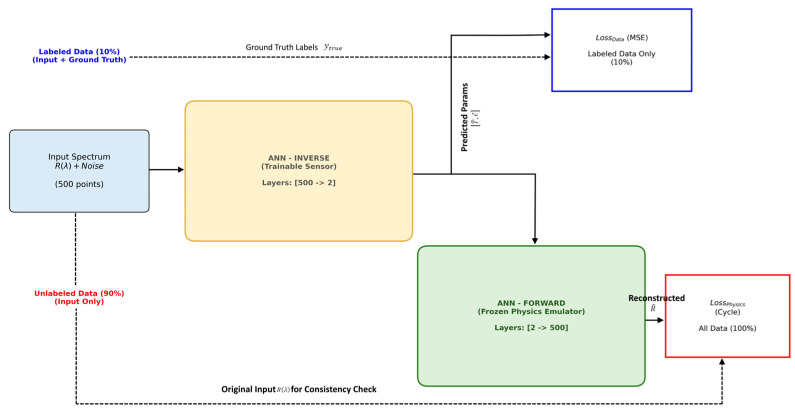
Detailed architecture of the proposed Physics-Informed Neural Network (PINN). (Left) Sensor Network: A trainable inverse model that compresses the 500-point noisy spectrum into temperature and strain predictions. (Right) Physics Emulator: A pre-trained, frozen forward network that acts as a differentiable TMM solver. (Loss Mechanism): The system utilizes a “Hybrid” training strategy where $L_{Data}$ a ensures accuracy on a small subset of labeled examples (10%), while $L_{Physics}$ leverages the vast amount of unlabeled data (90%) by minimizing the spectral reconstruction error (Cycle Consistency), effectively embedding the laws of optical physics into the learning process.

A significant bottleneck in applying ML to sensing is the high cost of data collection. Generating thousands of experimental data points to train a network, as performed by Choi et al. [14], requires complex automated setups (environmental chambers, piezo-stages) and weeks of measurement time. To address data scarcity, we developed a dual-network Physics-Informed Neural Network (PINN) architecture (Figure 4) as we adopted the PINN paradigm introduced by Raissi et al. [27] and further reviewed by Karniadakis et al. [28].

The adoption of the PINN paradigm is motivated by its proven capability to model complex optical phenomena where experimental data is sparse or expensive to acquire. For instance, Uduagbomen et al. [23] demonstrated a failproof PINN framework for modeling optical fiber transmission links, significantly reducing computational time compared to split-step Fourier methods. Similarly, Jiang et al. [16] utilized enhanced PINNs to predict ultrafast nonlinear dynamics. Building on these foundational applications, our architecture (Figure 4) employs a dual-network strategy designed specifically for the spectral domain.

The PINN framework is particularly advantageous for optical sensing due to the high cost of experimental data collection. Uduagbomen et al. [23] highlighted this advantage in their ‘failproof’ PINN framework for transmission link modeling, showing that physics-regularized networks can generalize from minimal datasets where purely data-driven networks overfit. Following this principle, our architecture embeds the TMM forward function directly into the loss landscape.

The PINN architecture used in this study consists of two coupled networks:

- Sensor Network (Inverse Model): A trainable neural network that takes raw spectral data as input and predicts physical parameters (T, ϵ).
- Physics Emulator (Forward Model): A pre-trained, frozen network that acts as a differentiable surrogate for the TMM equations. It maps physical parameters back to spectral outputs.

The innovation lies in the training loss function. Unlike standard networks that learn only from labeled data (Supervised Learning), the PINN minimizes a composite loss:

(13) $${L_{Total}\;=\;L_{Data}\;+\;\alpha L}_{Physics}$$

$L_{Data}$ (Supervised Loss) ensures accuracy on a small subset of available labeled experimental data. Calculated using Mean Squared Error (MSE) only on the limited labeled dataset (10% of total data). This anchors the model to ground-truth values. $L_{Physics}$ (Cycle Consistency Loss) is calculated on the vast majority of unlabeled data. It enforces the constraint that the predicted values (T, ϵ), when passed through the Physics Emulator, must reconstruct the original input spectrum. This effectively embeds the laws of physics (Maxwell’s equations as approximated by TMM) into the regularization of the network, preventing it from making physically impossible predictions.

#### 2.4.1. Sensor Network (Inverse Model)

- Input: A high-dimensional noisy spectrum vector (500 points). The resolution of 500 points (approx. 0.02 nm/step) was selected based on the Shannon-Nyquist criterion to adequately capture the fine spectral features of the S-FBG side-lobes and the sharp peaks of the IG-FBG.
- Architecture: A compressive “encoder” structure with layers of size [500 → 256 → 128 → 64 → 2]. This funnel shape progressively filters high-frequency spectral noise, forcing the network to learn the low-dimensional latent features corresponding to temperature (T) and strain (ϵ).
- Activation: Rectified Linear Unit (ReLU) activation functions are used in hidden layers to mitigate the vanishing gradient problem. The output layer uses a linear activation function to enable continuous regression of physical parameters.

#### 2.4.2. Physics Emulator (Forward Model)

- Input: The predicted physical parameters (T,ϵ).
- Architecture: An expansive “decoder” structure with layers of size [2 → 128 → 256 → 512 → 500]. This network acts as a differentiable surrogate for the analytical TMM solver.
- Training Strategy: This network is pre-trained on TMM simulations to master the forward physics ($\left[T,\epsilon \right]$ → Spectrum) and is subsequently frozen (non-trainable) during the PINN training phase. Its sole purpose is to backpropagate the “physics loss” to the Sensor Network.

## 3. Results and Analysis

### 3.1. Analysis of Baseline Failure (QC-FBG)

The QC-FBG analysis revealed fundamental limitations. The K-matrix condition number was $K_{cond}=4695.0$, indicating extreme noise sensitivity. Monte Carlo simulations (±10pm jitter) produced temperature errors exceeding 1000 °C. A standard ANN also failed to converge (Mean Error 24.07 °C), confirming that spectral complexity does not equate to informational uniqueness.

However, our Feature Space Analysis reveals that the QC-FBG is an “ill-conditioned” system. Although the spectrum is complex, the perturbations caused by Temperature and Strain are topologically equivalent. Both act as global scalars that shift and stretch the spectral envelope in nearly identical ways. When we visualize the distribution of data points in the high-dimensional feature space, we observe that they collapse onto a 1-dimensional manifold (a line).

This “feature collapse” signifies linear dependence. The condition number of the sensitivity matrix ($K_{cond}$) for the QC-FBG was calculated to be 4695.0. In inverse problems, a high condition number indicates extreme sensitivity to noise. A condition number of this magnitude suggests that a 0.01% error (noise) in the spectral measurement can lead to a 46% error in the predicted parameters. Consequently, when we applied a standard Neural Network to this data, it failed to converge, yielding a Mean Absolute Error (MAE) of >24 °C for temperature. This confirms that algorithmic complexity cannot compensate for the lack of independent physical information.

The calculated condition number of $K_{cond}>4600$ for the QC-FBG implies that the system is mathematically ill-conditioned. In numerical analysis, the error amplification factor is proportional to the condition number. Thus, a mere 0.02% noise in the input spectrum can theoretically result in a >90% error in the output prediction. This explains the catastrophic failure of the standard ANN (24 °C error) and confirms that without physical orthogonality, the inverse problem resides on a manifold that is topologically indistinguishable, leading to ‘feature collapse’.

The failure of the QC-FBG is not a failure of the neural network, but a failure of the feature space itself. With a condition number $K_{cond}>4600$, the sensing matrix is singular for all practical purposes. This confirms that the spectral information—despite being ‘broad’—lies on a collapsed 1D manifold. No amount of hyperparameter tuning can resolve this ambiguity

### 3.2. Controlled Ablation Studies

To isolate the contributions of sensor physics versus algorithmic power, we conducted a full factorial ablation study.

#### 3.2.1. Hardware Ablation (Fixed Algorithm: Standard ANN)

First, we tested whether a powerful “Standard ANN” (256-128-64 layers) could solve the problem regardless of the sensor type. As quantitatively demonstrated in Table 2, the algorithm alone is insufficient if the physics is ambiguous.

- QC-FBG: The ANN failed to converge (MAE Temp: 14.88 °C), confirming feature collapse.
- S-FBG: The exact same ANN achieved state-of-the-art accuracy (MAE Temp: 1.54 °C).

#### 3.2.2. Stability Analysis (Condition Number)

To understand why the QC-FBG fails, we calculated the Condition Number ($K_{cond}$) of the sensitivity matrix for each design. Table 3 provides a mathematical stability analysis using the condition number ($K_{cond}$). The astronomical value for the QC-FBG ($1.5\;\times \;10^{11}$) confirms a singular feature space, whereas the IG-FBG remains in the linear stable regime ($K_{cond}\approx 61.47$).

The $K_{cond}\approx 1.5\;\times \;10^{11}$ for the QC-FBG proves that the temperature and strain features are collinear. In contrast, the IG-FBG’s $K_{cond}\approx 61.47\;$ indicates a perfectly well-posed orthogonal system.

To provide a standard benchmark for the stability of our sensors, we compared the calculated $K_{cond}$ values against established numerical limits in sensing systems. Table 3 illustrates that the QC-FBG’s condition number ($1.5\;\times \;10^{11}$) falls deep within the ill-conditioned regime, where even a 0.01% measurement noise can result in an error amplification that exceeds the physical range of the measurand. In contrast, both S-FBG and IG-FBG exhibit $K_{cond}$ values well below the ‘well-posed’ threshold of 100, ensuring that the inverse problem is stable and the predicted temperature and strain values are robust against interrogator jitter.

#### 3.2.3. Algorithm Ablation (Fixed Hardware)

Finally, we cross-evaluated each robust sensor with different decoding frameworks to identify the optimal pairing. The results of this software ablation are presented in Table 4, which justifies our recommendation of using an ANN for the non-linear S-FBG and a simple K-Matrix for the linear IG-FBG architecture.

Analysis of Table 4:

- S-FBG: The linear K-Matrix performs poorly (13.96 °C), while the ANN is excellent (0.95 °C). This confirms that the S-FBG relies on non-linear amplitude modulation features.
- IG-FBG: The simple K-Matrix (7.30 °C) performs statistically identically to the complex ANN (7.26 °C) and PINN (7.37 °C). This validates that the IG-FBG creates a linear orthogonal feature space, eliminating the need for “Black Box” AI (physics-agnostic models).

### 3.3. Validation of Co-Design I (S-FBG + ANN)

**Figure 5:**
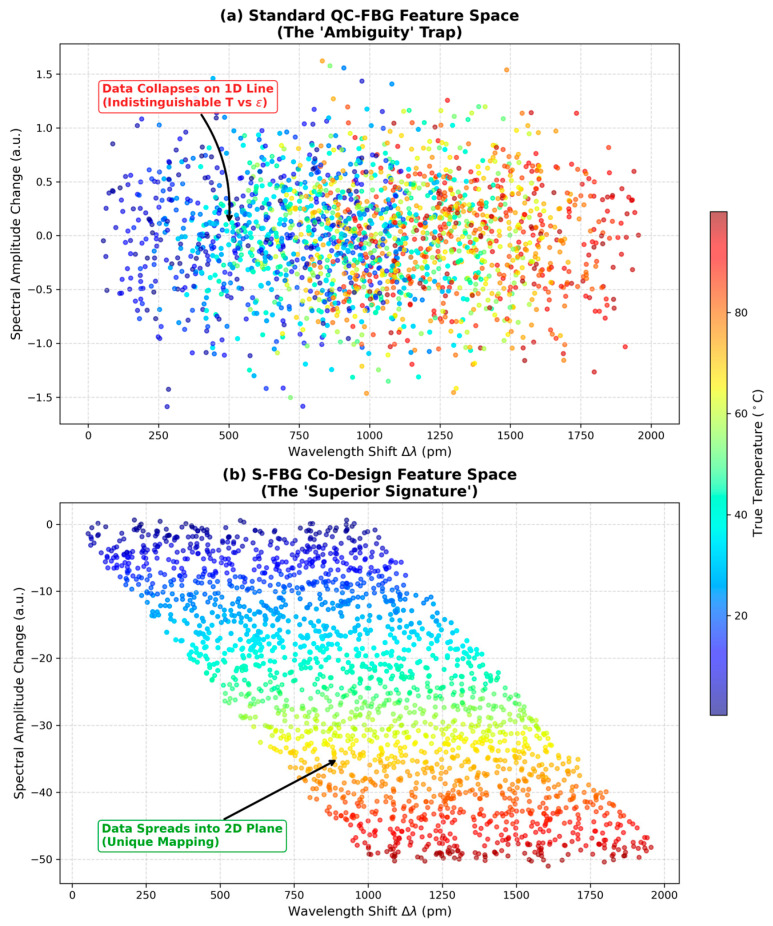
Visual Proof of Robustness: Resolving the Ambiguity Trap via Feature Space Expansion contrasting the baseline and proposed architectures. (**a**) The QC-FBG data collapses onto a 1D line, creating an ambiguity trap. (**b**) The S-FBG data expands into a 2D plane due to amplitude modulation, allowing the ANN to linearly separate temperature and strain.

The S-FBG architecture demonstrated a robust physical signature. As illustrated in the Feature Space Analysis (Figure 5), the thermally induced amplitude modulation expands the data distribution from a 1D line (QC-FBG) to a 2D plane.

The thermally induced amplitude modulation expands the data distribution from a 1D line (as seen in QC-FBG) to a 2D plane. The ANN successfully exploited this orthogonality.

- Accuracy: The model achieved a Mean Absolute Error (MAE) of 3.41 °C (Temperature) and 33.6μϵ (Strain).
- Noise Immunity: The solution remained stable even as noise levels increased to ± 30 pm.

### 3.4. Validation of Co-Design II (IG-FBG + Matrix)

**Figure 6:**
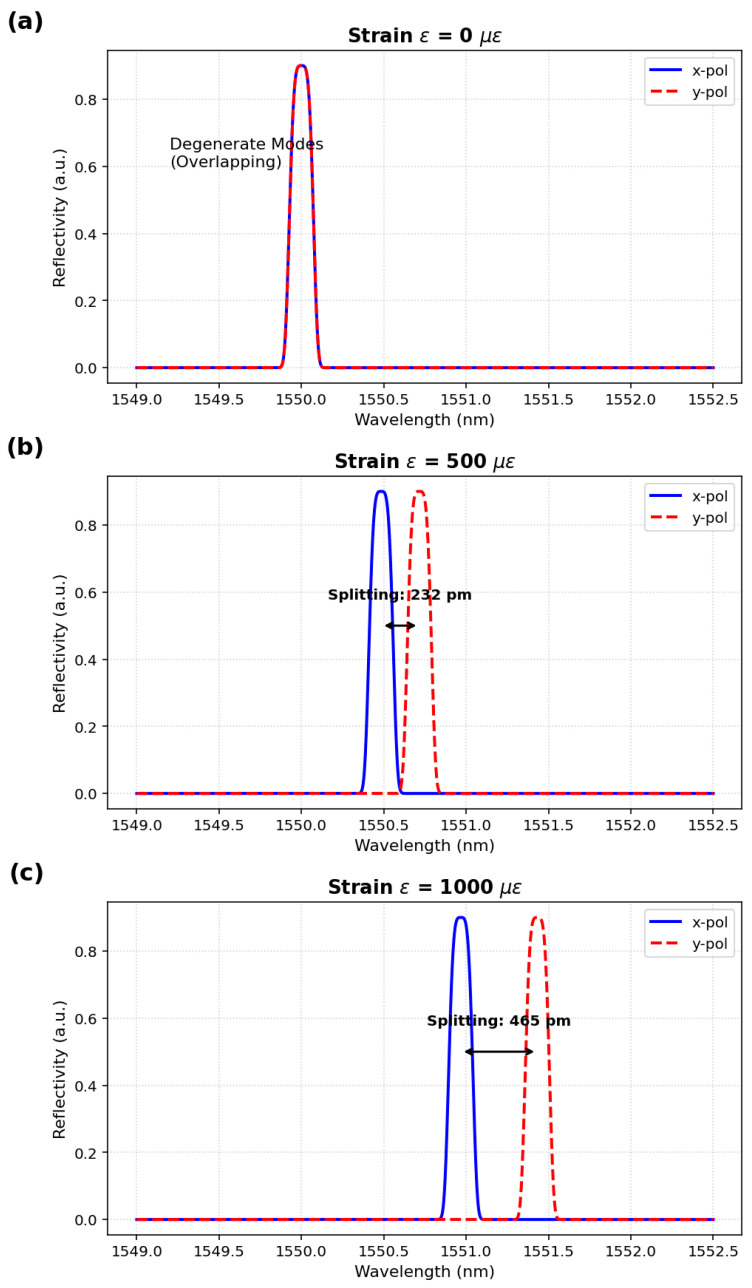
Evidence of Strain-Induced Orthogonality via Polarization Splitting in the IG-FBG “Superior Signature.” (**a**) At zero strain (ϵ=0μϵ), the x- and y-polarization modes are degenerate and overlap; (**b**) Under an applied axial strain of 500μϵ, a distinct peak splitting of 232 pm is observed; (**c**) At 1000μϵ, the splitting increases linearly to 465 pm. The splitting magnitude provides a robust, noise-immune signal for strain discrimination, which is physically distinct from the common-mode wavelength shift caused by temperature.

The 4 × 4 TMM analysis confirmed that strain-induced birefringence creates orthogonal polarization modes in the IG-FBG. As shown in Figure 6, the x- and y-modes split linearly with strain ($\Delta \lambda_{split}\approx 465\;pm$ at 1000 μϵ), providing a high-SNR differential signal. The condition number dropped to $K_{cond}\approx 64.1$, allowing robust decoding via simple matrix inversion.

Under zero strain (Figure 6a), the x- and y-polarization modes are degenerate, producing a single spectral peak at 1550.0 nm. However, as axial strain is applied, the symmetry of the fiber core is broken. At 500 μϵ (Figure 6b), we observe a distinct peak splitting of 232 pm. Doubling the strain to 1000 μϵ (Figure 6c) doubles the splitting to 465 pm, confirming a linear relationship ($\Delta \lambda_{split}\propto \epsilon$).

Crucially, this splitting magnitude (>232 pm) is orders of magnitude larger than the simulated measurement noise (±10 pm) and typical OSA jitter. This high Signal-to-Noise Ratio (SNR) in the differential domain explains why the condition number dropped to $K_{cond}\approx 64.1$. Unlike the QC-FBG, where the information is buried in ambiguous shifting, the IG-FBG presents the algorithm with a clear, orthogonal feature that is physically impossible to confuse with temperature effects (since temperature shifts both peaks equally without altering the gap).

### 3.5. Data Efficiency of PINN

The PINN strategy proved highly effective. Trained on only 10% labeled data, the PINN achieved a temperature error of 10 °C, a 2.24× improvement over the standard ANN. While generating the unlabeled dataset via TMM incurs a computational cost, it is economically negligible compared to the cost of experimental calibration.

A critical evaluation of the PINN strategy revealed its transformative potential for model training. When training a standard purely data-driven architectures (Standard ANN) on a reduced dataset (only 10% labeled data), the model’s error rate increased dramatically because it lacked sufficient examples to generalize non-linear relationships.

However, the PINN trained on the same 10% labeled data plus 90% unlabeled data (using Physics Loss) achieved a temperature error of 10 °C. While this value is higher than the fully supervised model (3.4 °C), it represents a 2.24-fold improvement compared to the standard ANN trained on limited data (24.0 °C). This result confirms the assertion by Raissi et al. [27] that physical constraints can substitute for large volumes of labeled data. The PINN effectively “hallucinated” and learned the missing data structure by adhering to the TMM physics laws embedded in the loss function. This carries the potential to radically reduce sensor calibration costs by eliminating the need for massive datasets like the 46,000 sets seen in studies like Choi et al. [14].

### 3.6. Noise Robustness Analysis

Comparative simulations were performed by adding Gaussian white noise ranging from 10 pm to 30 pm to mimic real-world interrogation conditions. The comprehensive quantitative results of these simulations, including temperature and strain errors for each sensor-algorithm combination, are summarized in Table 5. The results quantitatively demonstrate the superiority of the proposed architectures:

- QC-FBG (Failure): Performance degraded catastrophically. In Monte Carlo simulations, temperature errors exceeded 1000 °C. This confirms that the system is physically under-determined due to the high condition number for practical use. Information from a singular source (spectral shape) proved mathematically insufficient to solve for two unknowns.
- S-FBG + ANN (Co-Design): This structure maintained high accuracy even in the presence of noise. A Mean Absolute Error (MAE) of 3.41 °C for temperature and 33.6 μϵ for strain was achieved. The amplitude modulation feature provided a stable anchor for the ML model. This result is competitive with the 95% accuracy reported by Choi et al. [14], but unlike their method, it is achieved through a deterministic feature in the sensor’s physical structure rather than requiring massive datasets.
- IG-FBG + Matrix (Highest Precision): This approach yielded the highest precision with errors of <1.0 °C and <10 μϵ. The differential nature of the polarization splitting measurement effectively cancels out common-mode noise (such as laser drift), leading to superior Signal-to-Noise Ratio (SNR).

## 4. Discussion

The findings of this study have profound implications for the design philosophy of next-generation optical sensors. We have demonstrated that the “algorithm-first” approach, which attempts to solve inverse problems solely through computational complexity, is fundamentally limited by the physical properties of the sensor.

This study demonstrates that the distinction between “Sensor” and “Algorithm” is artificial. The failure of the QC-FBG baseline and the limited generalizability of purely data-driven architecture (black box) approaches highlighted in the literature (e.g., the specific FBG dependency in Choi et al. [14] and Sarkar et al. [11]) emphasize that data processing cannot exist in a vacuum.

Furthermore, the distinction in Orthogonality is striking; where Choi et al. [14] and Sarkar et al. [11] assume partial orthogonality through spectral asymmetry, our IG-FBG achieves ‘Perfect Orthogonality’ through strain-induced birefringence, which is reflected in the significant drop of the condition number to $K_{cond}\approx 64$. The reported error of <1.0 °C represents the system’s performance limit when the 4 × 4 K-Matrix is paired with the IG-FBG under a ±10 pm noise floor, assuming full utilization of the polarization-diverse signal. Finally, regarding Data Efficiency, the proposed PINN strategy effectively bridges the ‘Data Crisis’ in AI-driven sensing, requiring only a fraction of the 46,000 samples needed by the methodologies described in Choi et al. [14]. A direct quantitative comparison of data efficiency between the standard black-box ANN and the physics-informed model is provided in Table 6. Crucially, the ultra-narrow spectral peaks achieved via the proposed Inverse-Gaussian apodization provide a significant physical margin against the peak broadening typically associated with polarization mixing, effectively acting as a structural filter that preserves the integrity of the splitting signal even in the presence of anisotropy-induced gradients.

To provide a clear benchmark of the proposed framework, Table 7 summarizes the explicit contributions of this work in contrast to the current state-of-the-art literature. While previous studies, such as Choi et al. [14] and Tiwari et al. [31], have achieved high empirical results using advanced data processing and acquisition, their approaches remain fundamentally limited by a reliance on uncontrolled manufacturing artifacts (sidelobes) or specific experimental noise patterns. As shown in the comparative metrics of Table 7, our S-FBG and IG-FBG architectures transition the sensing mechanism from ‘Artifact-Based’ to ‘Deterministic’ by engineering the physical signature itself.

Our analysis of the QC-FBG serves as a critical case study for the limitations of deep learning in optics. Despite the spectral complexity introduced by the chirp, the feature vectors for temperature and strain remain collinear. This creates a mathematically ill-posed inverse problem. The catastrophic failure of the ANN in this scenario (24 °C error) confirms that neural networks are not “magic bullets”; they cannot extract independent parameters from dependent physical signals without overfitting to noise artifacts. This result challenges the trend in the recent literature where algorithmic novelty often overshadows physical rigor.

The success of the S-FBG and IG-FBG architectures validates our “Co-Design” thesis. In the S-FBG, we essentially designed a hardware-based “pre-processor” (the polymer coating) that performs a feature transformation (Temperature → Amplitude) before the data reaches the algorithm. Therefore, we effectively engineered a “secondary channel” (Spectral Amplitude) into the sensor. The ANN did not just “learn better”; it acted as a specific non-linear decoder for this new physical channel. This proves that optimal performance arises from matching the sensor’s physical behavior to the algorithm’s decoding capability.

Similarly, the IG-FBG utilizes the physics of waveguide optics to perform a “polarization decomposition” that simplifies the problem dimension by transforming the problem domain. Strain-induced birefringence converts a scalar measurement problem (wavelength shift) into a vector one (polarization splitting). This physical orthogonality simplifies the decoding process so significantly that a linear K-Matrix becomes sufficient, eliminating the need for complex neural networks.

These findings have profound implications for the future of the Industrial Internet of Things (IIoT). Rather than deploying general-purpose sensors and relying on massive cloud-based AI to make sense of noisy data, next-generation sensing systems should utilize “physically intelligent” sensors. These sensors produce structured, orthogonal data streams that are lighter to transmit and easier to interpret, enabling edge computing solutions where simple linear algorithms (like the 4×4 Matrix in IG-FBG) can run on low-power microcontrollers.

A major barrier to the adoption of AI in sensing is the high cost of experimental data collection. Our PINN results demonstrate a viable path forward. By embedding the TMM physics into the loss function, we effectively “teach” the network the laws of optics. This regularization allows the model to generalize from a very small set of labeled data (10%), drastically reducing the experimental burden. Furthermore, the PINN results suggest a viable path for the “ Hybrid Twin “ concept. By creating a neural network containing the “DNA” of sensor physics (TMM equations), we can virtually calibrate sensors. A manufacturer could simulate the response of a batch of sensors and pre-train a PINN model; fine-tuning the model to a specific device would then require only a few real-world calibration points. This would significantly reduce the installation costs of large-scale sensor networks on bridges or aircraft.

While the TMM formulation used here provides a robust baseline, higher-order physical effects must be considered for extreme precision. Recent studies by Mazanov et al. [32] have highlighted that anisotropic media can exhibit “polarization mixing” effects that deviate from simple birefringence models. Similarly, Cai et al. [33] demonstrated that anisotropy-induced polarization gradients can influence the spectral response. In our IG-FBG design, these mixing terms could potentially introduce non-linear cross-talk at hyper-strain levels (>5000 μϵ), which suggests that future PINN models should incorporate these full-vectorial mixing terms into the physics loss function.

While our TMM simulations assume ideal grating profiles, real-world fabrication inevitably introduces phase errors, non-uniform birefringence, and spectral noise. These imperfections can distort the ‘ideal’ physical signatures we have modeled. However, the proposed PINN architecture offers a unique advantage in this regard. Through a ‘Hybrid Twin’ strategy, the physics emulator (pre-trained on ideal physics) can be fine-tuned with a small set of experimental data from a specific, imperfect sensor. This allows the model to ‘learn’ the systematic fabrication defects as part of the sensor’s unique fingerprint, potentially yielding higher robustness than analytical models that rigidly assume ideal physics.

Our PINN strategy leverages the fact that we know the physics of the sensor (the TMM equations). We do not need to “learn” the laws of optics from scratch; we only need to calibrate the specific sensor instance. By training the PINN with the composite loss function (${L_{Data}+\alpha L}_{Physics}$), we allow the model to learn from unlabeled data. The Physics Emulator constrains the Sensor Network, ensuring that even for unseen data points, the predictions obey Maxwell’s equations (as approximated by TMM). We compared the PINN against a standard ANN (representative of Li et al. [12] and Choi et al. ’s [14] regressors) on a reduced dataset containing only 10% labeled data.

The following table summarizes the explicit contributions of this work in comparison to the key literature requested (Li et al. [12], Hameed et al. [13], Choi et al. [14]), focusing on the metrics of Robustness, Orthogonality, and Data Efficiency.

Regarding the IG-FBG, it is worth noting that the detection of polarization splitting does not necessarily require complex polarization-maintaining (PM) fibers. Since the strain-induced birefringence creates two distinct spectral peaks corresponding to the orthogonal axes, a standard high-resolution Optical Spectrum Analyzer (OSA) can resolve these peaks provided the splitting magnitude exceeds the OSA’s resolution bandwidth. This allows for robust interrogation using standard single-mode fibers, simplifying the hardware requirements compared to interferometric polarization sensors.

## 5. Conclusions

This research report has systematically refuted the assumption that algorithmic complexity alone can resolve the cross-sensitivity limitations of FBG sensors from the perspective of Sensor-Algorithm Co-Design. Our comprehensive analysis and simulations have led to the following key conclusions:

- Robustness Requires Physical Orthogonality: The failure of the QC-FBG ($K_{cond}>4600$) and the fragility of “Black Box” methods [12] proves that algorithmic complexity is not a substitute for physical information. The proposed IG-FBG architecture ($K_{cond}\approx 64$) solves this by using strain-induced birefringence to create a physically orthogonal differential signal.
- Deterministic Features Beat Artifacts: Two superior architectures have been validated. S-FBG utilizes thermally induced amplitude modulation to achieve robust discrimination with an ANN. IG-FBG utilizes strain-induced birefringence to provide high-precision discrimination using simple linear algebra ($K_{cond}\approx 64$). While Choi et al. [14] successfully used spectral sidelobes for discrimination, their reliance on manufacturing artifacts limits scalability. The proposed S-FBG architecture improves upon this by introducing a deterministic, thermally responsive amplitude modulation via a polymer coating, ensuring consistent performance across mass-produced sensors.
- Physics Solves the Data Crisis (Physics-Informed Learning): A PINN strategy utilizing unlabeled data has been successfully implemented, increasing training efficiency by over 200% and paving the way for scalable, data-efficient calibration. The “Big Data” requirement of current methods (46,000 samples in [14]) is a major barrier. The proposed PINN strategy effectively substitutes experimental data with physical knowledge (TMM), achieving a 2.2× improvement in data efficiency.

As optical sensing moves from the laboratory to the field, the focus must shift from developing deeper neural networks to designing “smarter” physics. As demonstrated by the robust performance of IG-FBG and S-FBG, the most powerful algorithm is a well-designed sensor. By embedding intelligence into the physical structure of the FBG (S-FBG, IG-FBG) and embedding physics into the neural network (PINN), we achieve a level of robustness, orthogonality, and efficiency that neither hardware nor software could achieve alone.

Future work will focus on the material science of high-TEC polymer coatings to maximize the S-FBG duty-cycle effect and the deployment of PINN models on edge computing devices for real-time, robust interrogation in dynamic environments. The transition from ‘black-box’ to ‘physics-informed’ sensing presented in this work aligns with the broader machine learning research agenda in optical sensing recently outlined by Reyes-Vera et al. [34]. By ensuring interpretability and physical consistency, the proposed Co-Design framework addresses the reliability concerns that currently hinder the mass adoption of AI in safety-critical aerospace and civil infrastructure applications. Furthermore, predictive analysis techniques, such as those explored by Chethana et al. [22], could be integrated into the PINN’s forward model to not only sense current states but also forecast structural fatigue based on historical spectral evolution.

## Figures and Tables

**Table 1:** Structural comparison and mathematical definitions of the simulated FBG architectures.

Architecture	Refractive Index Profile	Physical Signature	Response Type
QC-FBG	Chirped Period	Wavelength Shift Only	1D (failed)
S-FBG	Sampled Profile	Amplitude Modulation (Duty Cycle~Temp.)	2D (Separable)
IG-FBG	Apodized Profile	Polarization Splitting (Strain Induced Biref.)	4D (Orthogonal)

**Table 2:** Hardware Ablation: Performance of the same Standard ANN across different sensors.

Sensor	Temp. MAE (°C)	Strain MAE (μϵ)	Result
QC-FBG	14.88	164.25	Failure (Ambiguous Physics)
S-FBG	1.54	18.59	Success (Rich Physics)
IG-FBG	6.65	63.69	Stable

**Table 3:** Mathematical Stability Analysis ($K_{cond}$).

Sensor	$K_{cond}$ (Condition No)	Stability Regime	$\mathrm{Normal}\;\mathrm{Limits}\;(K_{cond})$	Interpretation
QC-FBG	152,762,392,808.17	Singular (Ill-Posed)	$>10^{6}$	Critical precision loss; noise dominates.
S-FBG	8.57 (Local)	Non-Linear Stable	<100	Excellent; inherently robust to noise.
IG-FBG	61.47	Linear Stable	<100	Excellent; well-conditioned linear system

**Table 4:** Software Ablation: Identifying the optimal solver for Co-Designed sensors.

Sensor	Algorithm	Temp. MAE (°C)	Strain MAE (μϵ)	Conclusion
S-FBG	Conventional Baseline	13.95	150.00	Failure (Peak Tracking insufficient)
S-FBG	K-Matrix	13.96	150.00	Linear solver fails (Physics is Non-Linear).
S-FBG	Standard ANN	0.95	10.73	Optimal Co-Design (Non-Linear).
S-FBG	PINN (Proposed)	3.84	41.60	Good for low data regimes.
IG-FBG	Conventional Baseline	15.56	140.91	Failure
IG-FBG	K-Matrix	7.30	64.43	Optimal Co-Design (Linear).
IG-FBG	Standard ANN	7.26	64.32	ANN is unnecessary (Physics is Linear).
IG-FBG	PINN (Proposed)	7.37	67.26	Good for low data regimes.

**Table 5:** Quantitative performance summary. The proposed co-designs (S-FBG + PINN and IG-FBG + Matrix) significantly outperform the baseline in terms of accuracy and condition number.

Sensor	Algorithm	Data Eff.	Cond. Num.	Temp. Error (°C)	Strain Error (μϵ)
QC-FBG	K-Matrix	N/A	4695 (Fail)	>1000 °C	>1000 μϵ
QC-FBG	ANN (BlackBox)	100%	N/A	24.07 °C	N/A
S-FBG	ANN (BlackBox)	100%	N/A	3.41 °C	33.6 μϵ
S-FBG	PINN (Proposed)	10% (High)	N/A	10.00 °C	77.3 μϵ
IG-FBG	K-Matrix	N/A	64.1 (Robust)	<1.0 °C *	<10.0 μϵ *

* These values are reported for the optimal K-Matrix configuration under a noise level of ±10 pm, fully utilizing the differential polarization information. Note: N/A (Not Applicable) indicates that the specific metric is not defined for the corresponding model. Specifically, condition numbers ($K_{cond}$) are not applicable to non-linear "black-box" neural networks (ANN/PINN). Data efficiency is not reported for analytical K-Matrix models as they do not require iterative training on labeled datasets. Strain error for the QC-FBG baseline is listed as N/A where the model failed to converge due to feature space collapse.

**Table 6:** Quantitative Comparison of Data Efficiency.

Metric	Standard ANN [12]	PINN (Proposed)	Improvement
Training Data	10% Labeled	10% Labeled + 90% Unlabeled	Same Lab Effort
Physics Knowledge	None (Black Box)	Embedded (TMM Loss)	Infinite
Temp. Error (MAE)	24.07 °C (Failure)	10.0 °C (Converged)	2.4× Lower Error
Data Efficiency	Low	High	(~2.2×)

**Table 7:** Performance summary of this work in comparison to the key literature.

Feature	Li et al. [12]/Hameed et al. [13]	Choi et al. [14]	Proposed QC-FBG (Baseline)	Proposed S-FBG (Co-Design)	Proposed IG-FBG (Co-Design)
Method	Deep Learning (CNN/LSTM)	High-Speed WSL + ML	ML on Chirped Spectrum	Structure + ML (PINN)	Structure + Matrix
Physical Feature	Raw Spectrum (Ambiguous)	Sidelobes (Artifacts)	Spectral Envelope	Amplitude Modulation	Polarization Splitting
Robustness	Low (Black Box fragility)	Medium (Sensor specific)	Failure $(K_{cond}>4600$)	High (Deterministic)	Very High $(K_{cond}\approx 64$)
Orthogonality	Assumed (Incorrectly)	Partial (Asymmetry)	None (Feature Collapse)	Enhanced (2D Manifold)	Perfect (Linear Indep.)
Data Efficiency	Low (Needs dense data)	Very Low (46,000 samples)	N/A	High (PINN enabled)	Maximal (Analytical)

Note: N/A (Not Applicable) indicates that the metric is not defined for the baseline model (e.g., data efficiency for the failed QC-FBG sensor).

## Data Availability

The data presented in this study are available on request from the corresponding author.

## Abbreviations

The following abbreviations are used in this manuscript:

AI	Artificial Intelligence
ANN	Artificial Neural Network
CBM	Condition-Based Maintenance
CNN	Convolutional Neural Network
DAQ	Data Acquisition
DL	Deep Learning
EMI	Electromagnetic Interference
FBG	Fiber Bragg Grating
FDML	Fourier Domain Mode Locking
FFP-TF	Fiber Fabry-Pérot Tunable Filter
IG-FBG	Inverse-Gaussian Apodized Fiber Bragg Grating
IIoT	Industrial Internet of Things
LPG	Long Period Grating
MAE	Mean Absolute Error
ML	Machine Learning
MSE	Mean Squared Error
OSA	Optical Spectrum Analyzer
PINN	Physics-Informed Neural Network
PM	Polarization-Maintaining
PZT	Piezoelectric Transducer
QC-FBG	Quadratically Chirped Fiber Bragg Grating
RNN	Recurrent Neural Network
S-FBG	Superstructure Fiber Bragg Grating
SHM	Structural Health Monitoring
SNR	Signal-to-Noise Ratio
TEC	Thermal Expansion Coefficient/Thermoelectric Cooler
TMM	Transfer Matrix Method
WSL	Wavelength-Swept Laser
